# Influence of the Resin Matrix Phase on the Fatigue Resistance of Model Dental Composite Resins

**DOI:** 10.3390/polym17233118

**Published:** 2025-11-24

**Authors:** Diana Leyva del Rio, Robert R. Seghi

**Affiliations:** Division of Restorative and Prosthetic Dentistry, The Ohio State University—College of Dentistry, 305 W 12th Ave., Columbus, OH 43210, USA

**Keywords:** composite, UDMA, Bis-GMA, fit852, viscoelastic, fatigue

## Abstract

This study aimed to assess how different resin matrix formulations affect the fatigue resistance of resin dental composites. Model dental composites were formulated using six distinct monomer mixtures: two Bis-GMA (bisphenol A-glycidyl methacrylate):TEGDMA (triethylene glycol dimethacrylate) (60:40 and 80:20 mole%), two UDMA (urethane dimethacrylate):TEGDMA (60:40 and 80:20 mole%), one Bis-GMA:UDMA:TEGDMA (35:35:30 mole%), and one Fit852:UDMA:TEGDMA (35:35:30 mole%). Cyclic fatigue resistance (CFR) of the resin composites was measured in a biaxial test mode using staircase analysis. Additional evaluations included biaxial flexural strength (BFS), degree of conversion (DC), water sorption (WS), and viscoelastic properties of the unfilled resins, such as the storage modulus (E′), loss modulus (E″), tan δ (E″/E′), and stiffness (k′). Data were subjected to one-way ANOVA with Tukey post hoc analyses. Pearson correlation and stepwise regression analyses were conducted to examine the relationships among variables. The UT6040 model composite exhibited the highest CFR (82.61 ± 8.83 MPa), significantly outperforming other formulations. Tan δ of the resin matrix showed the strongest correlation with CFR (r = 0.974), and was also shown to be the most influential predictor for the CFR of the particulate composites. The composition of the resin matrix has a significant impact on the CFR of dental composites. Among the properties evaluated, the viscoelastic parameter tan δ emerged as a strong and reliable predictor of CFR, emphasizing the importance of targeting viscoelastic behavior in the design of dental composite formulations.

## 1. Introduction

Dental composite restorations are one of the most common procedures performed by general dentists. It is estimated that over 76 million composite restorations are placed each year in the US, accounting for approximately 60% of all direct posterior restorations [1]. It has been estimated that more than 55% of all restorations placed involve the replacement of existing failed restorations [2], suggesting there is a need to improve clinical performance. While posterior composite restorations can fail in any number of ways, secondary caries, bulk fractures, marginal ridge fractures, and wear all contribute to reasons for restoration replacement [2,3].

Overall, posterior composites experience a clinically acceptable annual failure rate of 1 to 3% percent in adult populations [4,5,6,7]. However, failure rates are not uniform across all posterior restorations. Rates of failure have been shown to vary based on several factors, including the size and location of the restoration placed [8,9] as well as the caries risk of the patient [5]. Some short-term studies show fracture as the main reason for failure, while longer-term studies exhibit secondary caries as the major reason for failure and fracture as the second most common reason [10]. Regardless, bulk fractures and marginal ridge fractures continue to be important reasons for composite failures in both long- and short-term clinical trials [5].

It has been suggested that fatigue may be the most important property for dental materials exposed to periods of cyclic loading while chewing food [11]. Fatigue is a process that involves the nucleation, propagation, and coalescence of cracks. Failures due to fatigue can manifest themselves in dental restorations as wear, fractured margins, delaminated layers, and bulk fractures [12]. Fatigue damage is likely to originate from small existing flaws in the material that progress over time from repeated masticatory forces [13]. The resulting damage leads to the accumulated degradation of a material over time, which is the result of the repeated applied stresses that are well below the critical failure stress but continue the growth of subcritical cracks into an ultimate catastrophic failure [14]. For particulate-reinforced resin composites, several mechanisms may participate in the fatigue process, including matrix cracking, matrix deformation, void formation, multidirectional cracking, filler debonding, and filler failure [12]. Therefore, composite fatigue remains a very important property to consider in composite material design.

The filler component of dental composites has made a significant impact on improving the physical and mechanical properties of the material. Filler packing density improves polymerization shrinkage, elastic modulus, and flexural strength [15,16,17]. A positive correlation between filler content and fracture toughness has also been reported [18,19]. The filler component of composites has been shown to significantly influence fatigue resistance, with a maximum effect being reached between 60 and 80 wt% fillers [20]. However, the influence of filler volume on most properties, including strength and toughness, is limited, and volumes exceeding approximately 60 vol% do not result in appreciable improvements and can even be detrimental to some properties [17,21]. Filler characteristics have also been shown to influence the fatigue resistance of resin composites. Ornaghi et al. [22] evaluated the cyclic fatigue resistance of experimental formulations with four different size distributions. In this study, the experimental group with the smallest filler size composition showed a statistically increased cyclic fatigue mean value compared to the experimental group with the biggest filler size composition. The authors mention that their results could be explained due to experimental composites with a smaller filler size display a higher dissipation of energy and delay in recovery that resulted in a delay in the fatigue fracture. Belli et al. [23] analyzed the fatigue behavior of commercial composites and observed that superior fatigue performance was observed in the composite with the highest filler fraction. The author concluded that lower strength degradation after cyclic fatigue in dental composites with higher filler fraction could be due to the susceptibility of the resin phase to both strain challenges and environmental degradation.

Dental composites are known to exhibit viscoelastic behavior when stress is applied due to the viscoelasticity nature of the resin matrix phase. Dynamic mechanical analysis techniques have been used to measure the viscoelastic properties of dental composites under a fixed sinusoidal stress that simulates the masticatory cycle. Mesquita et al. [24] evaluated the viscoelastic properties of four experimental groups with varying filler fractions. It was observed that a positive correlation existed between the elastic response and the filler fraction of commercial composites, in which higher-filled composites showed higher elastic moduli. Papadogiannis et al. [25] and Vaidyanathan et al. [26] observed significant differences in the viscoelastic response between commercial composites with different particle sizes. Masouras et al. [27] and Kaleem et al. [28] observed significantly higher elastic moduli and better resistance to creep deformation in dental composites with irregular shape fillers when compared to composites with spherical fillers.

Although the influence of fillers on the viscoelastic response of dental composites has been well examined, the relationship between the viscoelastic properties of the resin phase on the overall influence on the fatigue resistance of dental composites has not been fully explored. To date, much of the literature has focused on the evaluation of commercial composites. However, because these materials vary greatly in composition, it is difficult to evaluate the influence of any one component on the overall effect on the fatigue behavior. In this study, we used experimental resin formulations to examine the relationship between resin matrix viscoelastic behavior and its effect on the cyclic fatigue behavior of filled composite resins.

The aims of this study were:

1. To observe how the different base composition of the resin matrix phase of the particulate-filled composites affects properties such as BFS, CFR, DC, and WS.
2. To observe how the different base compositions of the neat resins affect their viscoelastic properties (E′, E″, tan δ, and k′).
3. To observe if the properties BFS, DC, and WS of the particulate-filled resins and the viscoelastic properties (E′, E″, tan δ, and k′) of the neat resins have any linear relationship with the CFR of the particulate-filled resins, and if these evaluated properties could be used to predict the fatigue resistance of the filled composite materials.

To provide an immediate overview of our research approach, this study investigated the influence of different resin matrix formulations on the fatigue resistance of model dental composite resins and other mechanical properties. We formulated six distinct monomer mixtures, including variations of Bis-GMA:TEGDMA, UDMA:TEGDMA, Bis-GMA:UDMA:TEGDMA, and an experimental Fit852:UDMA:TEGDMA blend, all incorporating specific photo-initiators and accelerators with identical silanated fillers. Our methodology involved comprehensive evaluations of the resulting composites, measuring cyclic fatigue resistance (CFR) through a biaxial test mode using the “staircase” analysis, along with biaxial flexural strength (BFS), degree of conversion (DC), and water sorption (WS). Furthermore, the viscoelastic properties (storage modulus E′, loss modulus E″, tan δ, and stiffness k′) of the unfilled resins were assessed using dynamic mechanical analysis. This systematic experimental design allowed for a detailed examination of how the resin matrix composition impacts the performance of dental composites, with statistical analyses employed to identify key relationships and predictive factors. 

The null hypotheses of this study were: (1) The base composition of the resin matrix phase of particulate-filled composites does not significantly affect BFS, CFR, DC, and WS; (2) the base composition of the neat resin does not significantly influence its viscoelastic properties (E′, E″, tan δ, and k′); and (3) there is no significant correlation coefficient between the CFR of particulate-filled composites and the BFS, DC, and WS of the particulate-filled composites as well as the viscoelastic (E′, E″, tan δ, and k′) properties of the neat resins. Furthermore, these properties will not result in a significant model that could be used to predict the relative fatigue resistance of the particulate-filled composites.

## 2. Materials and Methods

### 2.1. Experimental Resin Formulations

Six experimental resin formulations were evaluated. Five of the formulations consisted of mixtures of traditional dental monomers. These included Bis-GMA (bisphenol A-glycidyl methacrylate):TEGDMA (triethylene glycol dimethacrylate) mixtures of 60:40 and 80:20 resin mole % ratios, UDMA (urethane dimethacrylate):TEGDMA mixtures of 60:40 and 80:20 resin mole % ratios, and a Bis-GMA:UDMA:TEGDMA mixture of a 35:35:30 resin mole % ratio. One additional formulation included a mixture that contained a high-molecular-weight experimental urethane-based, low-shrinkage resin (Fit852), and consisted of a mixture of Fit852:UDMA:TEGDMA at a 35:35:30 resin mole % ratio (Table 1). Additionally, CQ was used as a photo-initiator (0.5 wt%) and 2-(Dimethylamino)ethyl methacrylate (DMAEMA) was used as an accelerator (0.1 wt%) (Sigma–Aldrich Chemie, Munich, Germany). The experimental particulate-filled composites were fabricated using each of the experimental resin formulations and the addition of identical amounts of silanated fillers in a 30/70 wt% ratio (Table 1). The inorganic fraction constituted 90 wt% silanated barium borosilicate glass fillers (Esstech, Essington, PA, USA) and 10 wt% silanated fumed silica (Aerosil Ox-50).

### 2.2. Biaxial Flexural Strength

Disk-shaped specimens (15.0 mm diameter × 1.1 mm thickness) were fabricated for each experimental particulate-filled composite using a ring-shaped plastic mold. The top and bottom surfaces were covered with Mylar, compressed between 2 flat glass plates, and photoactivated for 40 s on each side at 677 mW/cm^2^ irradiance with a light-curing unit (Optilux Dementron, Kerr). The specimens were stored dry at room temperature for 24 h, to be later polished using a 600-grit silicon carbide powder on one side under copious water irrigation for a standardized time of 30 s. The specimen’s dimensions were recorded with a digital caliper. A spherical piston on a ring device was used to perform a biaxial bending test, where each specimen was placed concentrically on a multiple-ball ring device, and then a load was applied at the center of the specimen. Using a universal testing machine (Instron 5500R, Norwood, MA, USA), the specimens were loaded until fracture with a crosshead speed of 1 mm/min (*n* = 15). The BFS of each specimen was calculated using the following equation [29]:

$$\mathrm{BFS}\;=\;\frac{3P(1+v)}{4\pi t^{2}}\left[1+2ln\frac{a}{b}+\frac{\left(1-v\right)}{\left(1+v\right)}\left\{1-\frac{b^{2}}{2a^{2}}\right\}\frac{a^{2}}{R^{2}}\right]$$

where *P* is the load (in N); *t* is the disc thickness (in mm); *a* is the radius of the circle of support; *b* is the radius of uniform loading at the center; *R* is the radius of the disk; *v* is Poisson’s ratio (*v* = 0.30 for all composites).

### 2.3. Cyclic Fatigue Resistance

To evaluate the cyclic fatigue resistance (CFR) of the experimental particulate-filled composites, the “staircase” approach was used. Additional disk-shaped specimens were fabricated. While submerged in water at ~36 °C (simulating normal body temperature), all specimens were subjected to 10^4^ cycles at a frequency of 2 Hz (*n* = 20) in an MTS testing machine in a biaxial test mode. Tests were conducted sequentially, with the maximum applied stress in each succeeding test being increased or decreased by a fixed increment of 5 MPa, according to whether the previous test resulted in failure or not. The maximum stress for the first specimen was empirically set to 50% of the BFS value. The CFR mean and standard deviation values of each group were calculated according to the following equations [30]:

$$\mathrm{CFR}\;=\;X_{0}+d\left(\frac{\sum {in}_{i}}{\sum n_{i}}\pm 0.5\right)$$

$$\mathrm{SD}=1.62d\left(\frac{{\sum n_{i\;}\;\sum i^{2}n_{i}-\left(\sum in_{i}\right)}^{2}}{{\left(\sum n_{i}\right)}^{2}}+0.029\right)\;\;\;\;\;\;\mathrm{if}\;\frac{{\sum n_{i\;}\;\sum i^{2}n_{i}-\left(\sum in_{i}\right)}^{2}}{{\left(\sum n_{i}\right)}^{2}}\;\geq 0.3$$

$$\mathrm{SD}=0.53d\;\;\;\;\;\;\;\;\;\;\;\;\;\;\mathrm{if}\;\frac{{\sum n_{i\;}\;\sum i^{2}n_{i}-\left(\sum in_{i}\right)}^{2}}{{\left(\sum n_{i}\right)}^{2}}<0.3$$

where *X*_0_ is the lowest stress level considered in the analysis and *d* is the fixed increment (5 MPa). To determine the CFR, the analysis of the data is based on the least frequent event (failures vs. non-failures). In the CFR equation, the positive sign is used when the analysis is based on non-failures; otherwise, the negative sign is used. For the analysis, the lowest stress level considered is designated as *i* = 0, the next as *i* = 1, and so on, and *n_i_* is the number of failures or non-failures at the given stress level.

### 2.4. Degree of Conversion

The degree of conversion of the experimental particulate-filled composites was evaluated using differential scanning calorimetry (DSC-Q100, TA Instrument Co., New Castle, DE, USA). Uncured particulate-filled composite (approximately 20 mg) of each group (*n* = 4) was placed in open aluminum DSC pans and covered with a 3D-printed cover that allowed the light-curing unit to irradiate the sample while covering the rest of the sample chamber. The distance between the tip of the light-curing unit and the sample was standardized at 5 mm. An additional empty aluminum pan was used as a control. Samples were irradiated 3 times, 1 min each time, with an additional minute between irradiations. Three exotherm peaks were recorded; the first corresponded to the heat generated by the polymerization process of the material plus the heat generated by the light-curing unit. The second and third peaks corresponded to the heat generated by the light-curing unit only. The area under each peak was integrated, and the total isothermal heat of the reaction was obtained by subtraction of the average of the last two peaks and the first peak [31,32]. The degree of conversion was calculated by correlating the heat flow of the polymerization process with the theoretical heat release per mole of the reacted carbon double-bonds (55 kJ/mol) [33].

### 2.5. Water Sorption

To evaluate water sorption, disk-shaped specimens (15.0 mm diameter × 1.1 mm thickness) were fabricated for each experimental particulate-filled composite using a ring-shaped plastic mold. The top and bottom surfaces were covered with Mylar, compressed between 2 flat glass plates, and photoactivated for 40 s on each side at 677 mW/cm^2^ irradiance with the light-curing unit. The specimens were stored in a desiccator with silica gel for 22 h at 37 °C, to be later transferred to a separate desiccator at room temperature for 2 h. The samples were regularly weighed using an analytical balance until a constant weight (m1) was obtained. The thickness and diameter of the samples were measured with a digital caliper, and the volume (V) was calculated in mm^3^. The specimens were then placed individually in glass phials that were fully immersed in 10 mL of distilled water, and weighed in a 24 h cycle until reaching equilibrium (m2). After reaching equilibrium, they were placed back into the desiccator and weighed every 24 h, repeating the same cycles required to obtain m1 until the mass equilibrium (m3) was reached. The following equation was used to obtain water sorption and solubility of the experimental particulate-filled composites: (m2 − m3)/V.

### 2.6. Dynamic Mechanical Analysis

To evaluate the viscoelastic properties (storage modulus [*E*′], loss modulus [*E*″], tan δ [*E*″/*E*′], and stiffness [*k*′]), rectangular beam-shaped samples (*n* = 4) of the experimental unfilled resin formulations were made using a custom-made steel mold (25 mm × 2 mm × 2 mm). The beam-shaped samples were fabricated by placing unpolymerized material in the mold and covering it with a Mylar strip, to then be photoactivated for 20 s with the light-curing unit overlapping the irradiation of segments of the sample. The samples were stored dry for 24 h, to later be polished using a 1000-grit silica carbide paper under copious water for a standardized time of 30 s. Before loading the samples into the DMA equipment, each sample was measured with a digital caliper, and the dimensions were entered into the equipment software prior to running the dynamic mechanical analysis. The samples were then subjected to dynamic mechanical testing (DMA-Q800, TA Instruments Co., New Castle, DE, USA) in the single-cantilever mode at an amplitude of 20 μm and frequencies of 1, 2, 3, 5, 7, and 10 Hz, representing a wide range of masticatory rates. The resulting output from each sample analysis provided raw values for *E*′, *E*″, tan δ, and *k*′. The data obtained at the different frequencies were then averaged to obtain a single value.

### 2.7. Statistical Analysis

A one-way ANOVA was used for the statistical analysis of each of the properties evaluated, including BFS, CFR, DC, WS, storage modulus (*E*′), loss modulus (*E*″), tan δ (*E*″/*E*′), and stiffness (*k*′). Pairwise comparisons between the experimental groups were assessed using Tukey post hoc tests. A Pearson correlation analysis was used to observe any linear relationship between CFR and the following variables: BFS, DC, WS, storage modulus (*E*′), loss modulus (*E*″), tan δ (*E*″/*E*′), and stiffness (*k*′). A stepwise regression analysis was evaluated using CFR as the dependent variable and using BFS, DC, WS, storage modulus (*E*′), loss modulus (*E*″), tan δ (*E*″/*E*′), and stiffness (*k*′) as the independent variables to observe if any of these variables are significant predictors of CFR. For all statistical testing, α = 0.05 was used. The statistical analysis was performed using statistical software SPSS (version 29, IBM; Armonk, NY, USA).

## 3. Results

The means and standard deviations of the measured BFS, CFR, DC, and WS are reported in Table 2. The superscript letters indicate groups that are significantly different (*p* < 0.05). The strength degradation values are also reported as the % reduction between the mean BSF and CFR values. For BFS, the highest value was obtained by group UT8020 (165.48 ± 12.27 MPa), which was not significantly different (*p* > 0.05) from groups UT6040 (163.78 ± 27.30 MPa) and FU3535 (164.12 ± 12.76 MPa). The BFS of the remaining three groups containing Bis-GMA in their resin formulations was significantly lower. For CFR values, the trend was similar. The highest CFR value was obtained by UT6040 (82.61 ± 8.83 MPa), which was not significantly different than UT8020 (82.06 ± 4.63 MPa), and again, the CFR values for the Bis-GMA-containing formulations were significantly lower.

**Figure 1:**
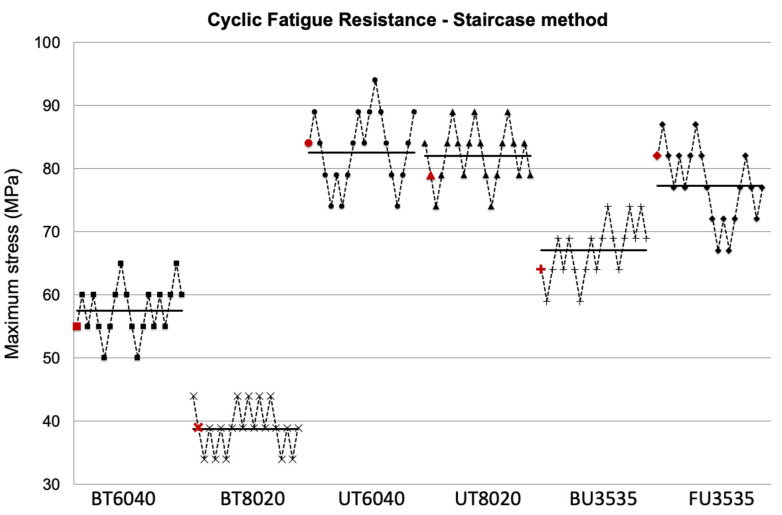
Cyclic fatigue resistance using the staircase method. Cyclic fatigue resistance results (in MPa) using the staircase method of the six experimental particulate-filled composites tested in this study. Horizontal lines represent mean values obtained in each group. Marks in red represent the samples before the first reversal of the staircase method.

Figure 1 represents the resulting plots of the staircase method data used to determine the CFR. The horizontal bars represent mean values, and the red markers represent the value before the first reversal in the staircase testing process, where the CFR analysis begins for each group. Although the CFR of group FU3535 was significantly lower than UT6040, it was still significantly greater than the other 3 Bis-GMA containing formulations.

All formulations resulted in a substantial amount of strength degradation, ranging from as low as 43.1% to a high of 55.1%. For the DC analysis, UT6040 obtained the highest value at 89.86%, which was significantly higher than the other experimental groups, while FU353 obtained the lowest DC value at 52.42%. For the WS analysis, UT8020 obtained the statistically lowest value at 22.10 (μg/mm^3^), while group FU3535 obtained the statistically highest value at 56.06 (μg/mm^3^). Table 3 summarizes the results of the measured viscoelastic properties of the unfilled resin mixtures. The means and standard deviations of four measured samples are reported. The superscript letters indicate groups that were statistically different. In general, the results of the elastic properties indicate that both UT6040 and UT8020 resulted in significantly higher storage modulus (*E*′) and stiffness (*k*′) values and significantly lower tan δ (*E*″/*E*′) values than the corresponding BT6040 and BT8020 formulations. Both tri-resin mixture formulations, BU3535 and FU3535, were found to be intermediate to these values. For the loss modulus (*E*″) of these resin formulations, UT8020 and FU3535 were significantly higher than the other groups, with BT6040 showing significantly lower values than all other formulations.

The Pearson correlation analysis (Table 4) revealed a positive correlation coefficient of 0.950 between CFR and BFS, as well as a significant correlation to the viscoelastic response of the neat resins, such as E′ and stiffness. However, tan δ of the resin phase showed the highest correlation coefficient, with a negative correlation of 0.974 with the CFR. The stepwise regression analysis showed the independent variable tan δ of the resin phase as the single most influential predictor for the CFR of the particulate-filled composites with a <0.001 significance.

## 4. Discussion

The factors that influence the fatigue resistance of dental composites have been well discussed in the literature [12,14]. While high filler volume fractions contribute significantly to improved mechanical properties, the filler geometry, size range, and distributions can confound the overall effect, making product comparisons difficult. To examine and isolate the influence of the resin matrix phase, we used model composites with identical filler particle sizes, distributions, and loadings. The effects of the filler volume on composite strength and toughness have been shown to reach a maximum at between 55 and 65 vol%. Because materials can be more consistently manufactured with wt% formulations, the model composites were filled at 70 wt%, which corresponds to about 55 vol% of a typical BaSiO_2_-filled dental composite [21]. In this investigation, the influence of the resin matrix phase was significant. The urethane-rich compositions produced composites with significantly greater mechanical properties, particularly superior fatigue resistance, compared to the other common formulations.

The relationship between cyclic fatigue strength and flexural strength of the particulate resin composites in this investigation showed a very high degree of correlation. However, many studies have shown that dental composites with high flexural strength values do not necessarily perform well under fatigue stress [34,35,36]. The difference in results is most likely explained by the fact that many studies have used commercial products for their evaluations. Commercial products vary in both their filler characteristics and their resin matrix formulations. This variability of commercial compositions makes it a challenge to assess and compare the influence of specific components on the overall properties of dental composites. Changes to the resin matrix formulation alone in this investigation resulted in significant differences in flexural and cyclic fatigue strength properties.

In this study, we use composite resin formulations with identical filler systems to focus specifically on the influence of the organic matrix on resulting properties, with emphasis on cyclic fatigue life. For the resin matrix, we used formulations that are typical of those found in commercial composite resins. The resin formulation varied in its monomer/co-monomer ratios to further observe any effect these formulation changes within a system may have on the properties evaluated. For the simpler two-resin formulations, increasing the oligomer to diluent monomer ratio has different effects depending on the system. In the case of the Bis-GMA/TEGMA-based system, the mechanical properties generally decreased, while the degree of conversion was not significantly changed. For the UDMA/TEGMA-based groups, the properties generally stayed the same while the degree of conversion decreased slightly.

The evaluation of flexural strength provides important information related to a material’s capability to withstand force under tensile stress. The effect of the filler content on the flexural strength of both commercial and experimental resin composites as a function of filler fraction [17,19,21,37], filler particle size [38,39,40], and filler particle shape [41] has been well studied. Because the filler phase has such a dramatic effect on the overall strength of a composite resin, the influence of the resin matrix has been less examined in the literature. In this study, we tried to isolate the resin matrix component as the variable to understand its structural impact. The results showed superior flexural strength performance of the UDMA (UT6040, UT8020, and BU3535)-containing composite resin materials, while groups containing only the base monomer Bis-GMA obtained the lowest values. These results are consistent with other studies that have demonstrated a similar resin effect on flexural strength [42]. Other studies that evaluated non-filled homopolymers based on Bis-GMA and UDMA have also demonstrated the inferior capability of Bis-GMA to withstand flexural strength stress in comparison to UDMA [43,44,45].

The current literature has pointed out that the importance of the analysis of fatigue properties of dental composites in vitro may help to predict clinical outcomes [11,46]. In this study, we observed that all experimental composites experienced a decrease in strength (strength degradation), which ranged from 43% to 55% after the application of mechanical fatigue of 10,000 cycles of the simulated masticatory process. The highest strength degradation was obtained by group BT8020 with 55.1%, while group BU3535 obtained the lowest strength degradation at 43.1%. To function in the oral cavity, the material should maintain its mechanical and physical integrity for an extended period. The oral environment is a complex system that results in physical alterations to dental restorative materials, given that they are subjected to both chemical degradation and cyclic mechanical forces. Fatigue can be described as the mechanical degradation of a material below a critical failure stress and involves the growth of subcritical defects at subcritical loads. Normal masticatory forces are cyclic by nature, where repetitive loading to dental restorations can lead to subcritical crack propagation, causing a catastrophic failure [14,47]. There are several factors that can affect the fatigue response of dental composites. Hydrolysis is known to influence the mechanical properties of dental composites and can affect the resin matrix, the fillers, and/or the matrix–filler interface [48,49]. Additionally, water has been shown to influence the fatigue of dental composites [47,50,51,52]. This is most likely due to the formation of cracks from continuous stresses applied to the material, which allows water to enter cracks, leading to the hydrolysis of the polymer matrix and the matrix–filler interface [53].

In this study, we did not investigate the influence of long-term degradation on fatigue resistance. Polymers primarily degrade through hydrolysis, which can be accelerated by enzymes that are present in saliva and can follow water into the interface and polymer structure. In this study, UDMA-based formulations obtained the lowest water sorption values; however, the tri formulation containing Fit852 obtained significantly higher water sorption values than the remainder of the groups. Water intake into polymers can shorten the lifespan of resin composites by causing silane hydrolysis and the formation of microcracks. Excessive water absorption may lead to the breakdown of the bond between silane and filler particles, resulting in filler–matrix debonding or even hydrolytic degradation of the fillers [54]. Gajewski et al. [44], in a study performed in unfilled homopolymers, obtained lower water sorption values of UDMA resin in comparison to Bis-GMA. Similarly, in our study, we found UDMA-based filled resins had lower water sorption than Bis-GMA-based filled resins. On the other hand, Fonseca et al. [55] found equal values for water sorption between Bis-GMA- and UDMA-filled resins. In the mentioned study, a filler resin formulation based on Fit852 was also assessed and, similar to our findings, exhibited significantly higher water sorption values. Although the exact structure of the monomer Fit852 is unknown, it is speculated that the monomer likely contains long, flexible urethane chains. When combined with TEGDMA, it may lead to a three-dimensional network structure with increased heterogeneity. In highly heterogeneous polymer networks, the variation between regions of high and low crosslink density can create larger voids, which are capable of absorbing greater amounts of water, resulting in higher water sorption values [56].

The analysis of DC is crucial for determining the properties of dental composites, performance, and biocompatibility with soft oral tissues. In this study, the highest DC values were obtained by two of the three Bis-GMA-free experimental composites (UT6040 and UT8020). It is relevant to mention that these two groups also yielded high values in the evaluated properties, such as BFS and CFR. Although group FU3535 obtained the lowest DC (52.42%), it also obtained high BFS values and good cyclic fatigue performance. Based on the results obtained in this study, the first null hypothesis was rejected since the base composition of the resin matrix phase of the particulate-filled composites does significantly affect the material’s properties tested here (BFS, CFR, DC, and WS).

This study also analyzed and compared the viscoelastic properties of the experimental unfilled resin formulations with the use of dynamic mechanical analysis. To the best of our knowledge, there is one single previous report in the literature that evaluates the viscoelastic properties of the resin matrix itself. Saen et al. [57] analyzed filled and unfilled dental resins under both static and dynamic mechanical testing. The authors fabricated formulations with a single resin matrix composition of 70 wt% Bis-GMA and 30 wt% TEGDMA as a co-polymer. Their results showed that the filled dental composites obtained higher storage modulus values and lower tan δ than their unfilled counterpart. In our study, we analyzed the viscoelastic properties of six resin matrix formulations with different base monomer and co-monomer ratios. Our results show that group BT8020 obtained the lowest storage modulus, representing how much energy a material can store when it is deformed elastically that will result in a low resistance to elastic deformation, and the highest tan δ, which represents the relative amount of energy dissipated versus the elastically stored in a material—and this group also obtained the lowest stiffness values from all the experimental groups. On the other hand, the experimental groups based on only UDMA (UT6040 and UT8020) obtained the highest storage modulus values, the lowest tan δ values, and the highest stiffness values. When analyzing filled dental composites, lower tan δ values would indicate a more elastic-like nature [58], which will result in a faster response from the restorative material to a load and a return to its original shape [24]. Therefore, the second null hypothesis was rejected since the base composition of the neat resin does significantly influence the viscoelastic properties tested here (E′, E″, tan δ, and k′).

We considered it necessary to observe if there was any linear association between the cyclic fatigue resistance of the particulate-filled composites and the viscoelastic behavior of the resin matrix, as well as other properties, such as the BFS, DC, and WS of the particulate resins. The Pearson correlation analysis showed significant correlations between the CFR and BFS of the particulate composite resins, as well as significant correlations between the viscoelastic response, such as the E′, tan δ, and stiffness of the neat resins. The stepwise correlation analysis showed the tan δ of the resin phase to be the most influential predictor for the CFR of dental resin composites. These results suggest that focusing on the viscoelastic response of the resin matrix in future dental composite design and testing could enhance fatigue resistance under cyclic loading, enabling a more targeted development of materials with improved long-term mechanical performance, particularly in posterior restorations where fatigue is a key concern. Based on the results obtained, we reject the third null hypothesis since there was a significant correlation coefficient between the CFR of the particulate-filled resins and the BFS, DC, and WS of the particulate-filled composites, as well as the viscoelastic (E′, E″, tan δ, k′) properties of the neat resins. Furthermore, these properties did result in a significant model that could be used to predict the relative fatigue resistance of the particulate-filled composites.

From the dimethacrylates commonly used in commercial composites, Bis-GMA is known to be highly viscous, mainly attributed to the presence of bulky aromatic groups, limiting the likelihood of the methacrylate groups coming into contact with each other and binding while showing a very rigid structure upon polymerization. For group BT8020, the high content of the stiff molecule Bis-GMA most likely caused a lower DC, which caused a negative effect on the mechanical properties. UDMA is another high-molecular-weight monomer that was introduced in commercial composites to overcome the limitations of Bis-GMA-based dental composites. It presents a very flexible backbone that results in lower viscosity and higher molecular flexibility [59], and it additionally forms strong intermolecular hydrogen bonds between urethane groups. Greater molecular flexibility of the monomers will directly affect their ability to form higher monomer-to-monomer conversion upon polymerization [60]. It has been demonstrated that UDMA monomer shows improved monomer-to-monomer conversion and polymerization kinetics than Bis-GMA [42,44], resulting in increased mechanical properties. This explains the higher values of certain properties, such as BFS and DC, for groups UT6040 and UT8020 when compared to the Bis-GMA-based groups (BT6040 and BT8020). Furthermore, in the analysis of the viscoelastic properties of the resin phase, these two UDMA groups obtained the highest storage modulus and lowest tan δ, demonstrating the capability of the system to store more energy. This can explain the higher fatigue resistance due to the experimental composite’s improved capacity to store energy within the polymer network, delaying a catastrophic failure.

Fit852 is a highly flexible, long-chain urethane-based methacrylate monomer with an absence of an aromatic group. This monomer has shown an increased degree of conversion when compared to Bis-GMA and UDMA [55], and lower shrinkage, which is claimed by the manufacturer. In a study by Manojlovic et al. [61], the Fit852:TEGDMA resin composites showed lower mechanical properties than the Bis-GMA:TEGDMA resin composites. In our study, we incorporated Fit852 into a UDMA and TEGDMA mixture. This group showed one of the lowest DC%; however, it had one of the highest flexural strength values and good fatigue resistance. This reveals that there are other characteristics in the final polymer network that compensate for the lower degree of conversion. However, further analysis of the molecule is needed to fully understand its properties to potentially incorporate it into mixtures for commercial products.

Some of the limitations of the present study include the need for a comparison of the viscoelastic properties of the formulations in their unfilled and filled forms. This could yield a further understanding of the effect of the fillers on the viscoelastic response of the material. Further studies could also focus on the effect of long-term water storage on the viscoelastic response of both unfilled resin formulations and particulate-filled composites. Another limitation of the current study is the need for a fractographic analysis using scanning electron microscopy (SEM) images. Such an analysis would have been useful to observe the fracture mechanisms and crack propagation pathways, providing deeper insights into the fatigue behavior of the model dental composite resins here evaluated.

## 5. Conclusions

Within the limitations of this study, it was found that the composition of the resin phase has an important effect on the fatigue resistance of experimental composites, as well as other mechanical and physical properties here tested. The viscoelastic property tan δ of the resin phase was found to be highly correlated to the CFR of the particulate resin composites. Similarly, tan δ of the resin phase was found to be the most influential predictor for the composite resin cyclic fatigue behavior. Furthermore, the novel monomer Fit852 may have some potential to be used in commercial formulations for dental composite resins with improved fatigue resistance.

## Figures and Tables

**Table 1:** Description of the composition of the experimental resins and particulate-filled composites used in this study.

Experimental Group	Resin Matrix Composition	Resin Mole % Ratio	Particulate Resin Composites wt% Ratio (Filler/Resin)
BT6040	Bis-GMA:TEGDMA	60:40	70:30 (Filler component: 90 wt% silanated barium borosilicate glass and 10 wt% silanated Ox-50)
BT8020	Bis-GMA:TEGDMA	80:20	
UT6040	UDMA:TEGDMA	60:40	
UT8020	UDMA:TEGDMA	80:20	
BU3535	Bis-GMA:UDMA:TEGDMA	35:35:30	
FU3535	Fit852:UDMA:TEGDMA	35:35:30	

**Table 2:** Means and standard deviations (in parentheses) of biaxial flexural strength (BFS, in MPa), cyclic fatigue resistance (CFR, in MPa) after 10^4^ cycles, fatigue strength degradation (in %), and degree of conversion (DC, in %) of the experimental composites used in the study.

Group Name	BFS (MPa)	CFR (MPa)	Strength Degradation (%)	DC (%)	Water Sorption (μg/mm^3^)
BT6040	102.96 (13.91) ^b,c^	57.50 (3.47) ^d^	44.2	61.73 (4.40) ^b,c^	28.20 (0.41) ^c^
BT8020	86.21 (11.60) ^c^	38.72 (2.65) ^e^	55.1	63.36 (9.43) ^b,c^	28.39 (0.44) ^c^
UT6040	163.78 (27.30) ^a^	82.61 (8.83) ^a^	49.6	89.86 (2.36) ^a^	24.68 (0.24) ^b^
UT8020	165.48 (12.27) ^a^	82.06 (4.63) ^a,b^	50.4	71.11 (13.52) ^b^	22.10 (0.87) ^a^
BU3535	117.89 (18.12) ^b^	67.06 (4.63) ^c^	43.1	58.96 (8.71) ^b,c^	25.40 (0.51) ^b^
FU3535	164.12 (12.76) ^a^	77.28 (9.43) ^b^	52.9	52.42 (3.56) ^c^	56.06 (0.80) ^d^

Values followed by the same superscript letter for a given property indicate no statistical differences (α = 0.05).

**Table 3:** Means and Standard deviations (in parentheses) of the viscoelastic properties of neat resins evaluated in the study. Storage modulus (*E*′) and loss modulus (*E*″) are represented in GPa, and stiffness (*k*′) is represented in N/m.

Group Name	Storage Modulus (*E*′)	Loss Modulus (*E*″)	Tan δ (*E*″/*E*′)	Stiffness (*k*′)
BT6040	0.66 (0.08) ^d^	0.147 (0.014) ^c^	0.224 (0.011) ^b^	1175 (150) ^d^
BT8020	0.63 (0.09) ^d^	0.166 (0.020) ^b^	0.263 (0.012) ^a^	1130 (167) ^d^
UT6040	1.83 (0.11) ^a^	0.179 (0.011) ^b^	0.098 (0.008) ^d^	3265 (201) ^a^
UT8020	1.85 (0.20) ^a^	0.193 (0.017) ^a^	0.105 (0.008) ^d^	3312 (350) ^a^
BU3535	1.17 (0.17) ^c^	0.177 (0.022) ^b^	0.152 (0.006) ^c^	2093 (300) ^c^
FU3535	1.37 (0.13) ^b^	0.200 (0.011) ^a^	0.147 (0.013) ^c^	2451 (234) ^b^

Values followed by the same superscript letter indicate no statistical differences (α = 0.05).

**Table 4:** Pearson’s correlation analysis.

		BFS	DC	WS	Storage Modulus (E′)	Loss Modulus (E″)	Tan δ (*E*″/*E*′)	Stiffness (*k*′)
CFR	Pearson Correlation	0.950 **	0.458	0.083	0.930 **	0.647	−0.974 **	0.930 **
	Sig. (2-tailed)	0.004	0.360	0.875	0.007	0.165	0.001	0.007

** Correlation is significant at the 0.01 level (2-tailed).

## Data Availability

The raw data supporting the conclusions of this article will be made available by the authors on request.
